# In Silico Identification of Novel Compounds as Anthelmintics Against *Haemonchus contortus* Through Inhibiting β-Tubulin Isotype 1 and Glutathione S-Transferase

**DOI:** 10.3390/ani15131846

**Published:** 2025-06-23

**Authors:** Yaqian Jin, Sheikh Arslan Sehgal, Faizul Hassan, Guiqin Liu

**Affiliations:** 1 Shandong Donkey Industry Technology Collaborative Innovation Center, College of Agriculture and Biology, Liaocheng University, Liaocheng 252000, China; jinyaqian@lcu.edu.cn; 2 Department of Genomics and Bioinformatics, Faculty of Animal Production & Technology, Cholistan University of Veterinary & Animal Sciences, Bahawalpur 63100, Pakistan; arslansehgal@cuvas.edu.pk; 3 Department of Breeding and Genetics, Faculty of Animal Production & Technology, Cholistan University of Veterinary & Animal Sciences, Bahawalpur 63100, Pakistan; f.hassan@cuvas.edu.pk

**Keywords:** anthelmintic resistance, β-tubulin mutations, glutathione S-transferase, in silico screening, bioinformatics, parasite biology

## Abstract

Haemonchosis caused by the parasitic worm *Haemonchus contortus* is a major threat to cattle and other ruminants and imposes significant economic losses in the livestock industry. Different medications have been reported; however, these are not reliable now due to mass drug resistance. The current study investigates potential inhibitors of two *H. contortus* proteins: glutathione S-transferase (GST) and beta-tubulin isotype 1. GST helps the parasite to detoxify harmful substances, while beta-tubulin is essential for the cell division and structure. By using computational approaches, natural compounds were identified to inhibit the selected proteins. The 3D structures of GST and β-tubulin isotype 1 were prepared, and pharmacophore models were generated to search the Molport natural compound library. The lowest binding energy ranged from −6.7 to −10.4 Kcal/mol. Post-docking interactional analyses revealed that Glu45, Arg46, Cys126, Gln131, Lys252, Asn247, and Arg251 residues were the most common interacting residues in β-tubulin isotype 1. Similarly, in GST, Leu99, Asn100, Arg103, Lys107, Glu162, and Met163 were the most common interacting residues. In conclusion, extensive computational analyses including virtual screening, docking, and MD simulations revealed that the compound Molport-039-195-358 might have the ability to control haemonchosis by targeting GST and β-tubulin isotype 1. The in silico studies identified potent compounds by targeting GST and β-tubulin isotype 1 against *Haemonchus contortus*. The reported findings provide a foundation for the development of novel anthelmintic therapies.

## Simple Summary

This study aims to identify novel compounds to control parasitic infestation caused by *Haemonchus contortus*, which is a major internal parasite of ruminants leading to huge economic losses. We identified natural compounds that can inhibit the two important enzymes of this parasite, leading to its death by hampering its detoxification system and disrupting the vital processes (cell division). This study reports a novel natural compound (Molport-039-195-358) with a potent ability to be used as an anthelmintic against *Haemonchus contortus.* Comprehensive in silico pharmacological and toxicological evaluations of the compound were carried out. It was observed that the reported compound is more potent compared to synthetic drugs owing to its ability to avoid drug resistance in the parasite. The present study offers a green solution to address parasitic infestation in ruminants to ultimately improve animal health and production.

## 1. Introduction

Haemonchosis is a disease caused by the parasitic worm *Haemonchus contortus*. Commonly referred to as the barber’s pole worm, *H. contortus* represents one of the most significant parasites that infects small ruminants, leading to substantial economic losses within the global livestock sector [1]. It is a frequent cause of mortality in cattle, goats, sheep, and other ruminants due to its blood-feeding behavior and the potential for the rapid development of large burdens [2]. Anthelmintic drugs are used widely to control *H. contortus.* However, the increased use of these drugs has led to severe drug resistance in animals [3]. Ivermectin and benzimidazole are widely used for the treatment of nematode infections; however, the unregulated and frequent use of ivermectin and benzimidazole has led to conditions that support the development of drug resistance in parasitic nematodes [4]. Due to the development of resistance in *H. contortus* against anthelmintic drugs, there is a need for new interventions to be used. Exploring possibilities for finding new antiparasitic drugs against *H. contortus* that have a role in the survival of the parasite could be a potential strategy.

Glutathione S-transferase (GST) and beta-tubulin isoform 1 are the key targets. GST is an important enzyme which is involved in the detoxification and protection of the parasite from anthelmintic drugs [5]. β-tubulin isotype 1 is a structural protein involved in cell division and intracellular transport and helps maintain the cytoskeleton of the parasite [6]. The regulation of these proteins may help control haemonchosis.

GSTs protect parasites from both toxic exogenous and endogenous substances through the facilitation of their conjugation to reduce glutathione [7]. GSTs are involved in drug resistance in parasites and neutralize the effects of anthelmintic drugs [8]. GST is considered a potential therapeutic target to control haemonchosis [9]. Thus, the inhibition of GST could be beneficial in hindering the detoxification systems of parasites.

β-tubulin isotype 1 is another significant protein of *H. contortus* and the target of benzimidazole-based drugs. These drugs are widely used to treat parasitic infections. β-tubulin isotype 1 is a key structural protein in *H. contortus*, and mutations in this gene have been linked to benzimidazole-class anthelmintic resistance. Benzimidazole resistance occurs due to mutations in the beta-tubulin gene that reduce the effectiveness of the drugs [10,11]. Beta-tubulin isoform 1 is involved in the formation of microtubules that are necessary for cell division and intracellular transport [12]. Hence, the inhibition of β-tubulin isotype 1 may disrupt these processes and lead to the death of the parasite.

*Haemonchus contortus* has shown resistance to anthelmintic drugs and become a serious issue in the health management of livestock worldwide. Imidazothiazoles and macrocyclic lactones, widely used drug classes, showed low efficacy due to rising resistance, and benzimidazoles interfere with the formation of microtubules by binding to β-tubulin. Macrocyclic lactones enhance the glutamate-gated chloride channels’ opening, leading to the death of the parasite. Levamisole targets the nicotinic acetylcholine receptors that cause spastic paralysis. The development of targeted therapeutics and multidrug resistance is essential to understand the mechanism.

Bioinformatics is an interdisciplinary field of science used to solve biological problems with the help of computational power, mathematics, and statistics [13,14]. Bioinformatics is used for in silico analyses to solve biological problems [15,16]. By using computational approaches, numerous drug-like compounds and inhibitors have been reported against diabetes, cancers, parasitic infections, and neurological diseases [11,17,18,19,20]. These approaches play an important role in finding drug-like compounds for disease targets. In silico approaches including molecular docking, pharmacophore modeling, ADMET analysis, and MD simulation are considered promising for finding inhibitors against different proteins involved in various diseases [20,21,22,23]. In this study, computational approaches were employed to identify potential natural compounds as inhibitors against β-tubulin isotype 1 and GST to mediate haemonchosis caused by *H. contortus.*

## 2. Materials and Methods

In the current analysis, protein structure prediction, pharmacophore modeling, pharmacophore-based virtual screening, molecular docking, ADMET analysis, and MD simulations were performed to screen potential inhibitors against GST and β-tubulin isotype 1. Numerous software and online tools, including PyRx [24], Discovery Studio [25], MODELLER 10.7 [26], UCSF Chimera 1.17 [27], PyMol 2.6 [28], I-TASSER [29], Robetta [30], and Desmond [31], were employed to find the potential inhibitors against the selected target proteins.

### 2.1. Structural Prediction and Protein Preparation

The 3D structure of β-tubulin isotype 1 from *H. contortus* was predicted through in silico analyses. The amino acid sequence of β-tubulin isotype 1 was retrieved from NCBI in FASTA format (accession number: ABM92348) consisting of 448 residues and subjected to the protein–protein Basic Local Alignment Search Tool (BLAST) to identify the suitable templates against the Protein Data Bank (PDB).

MODELLER 10.7 automated software was utilized to predict the 3D structure of β-tubulin isotype 1 through homology modeling approach by satisfying spatial restraints. I-TASSER, Rosetta, QUARK, and Robetta [29,32] were used to predict the 3D structures of the selected target proteins through threading and ab initio techniques. Numerous 3D structures of the selected target proteins were predicted through homology modeling, threading, and ab initio approaches, and the predicted structures were evaluated through different software including ERRAT, Anolea, RAMPAGE, ProCheck, Verify3D, WhatIF, and MolProbity. The outliers and poor rotamers were corrected by employing the WinCoot tool. The predicted structure was minimized by using UCSF Chimera 1.17 to ensure structural stability and to correct steric clashes [27]. The minimization was performed with 1000 steepest descent and 1000 conjugate steps by incorporating the Amber ff98 forcefield along with the conjugate gradient method.

The 3D structure of GST was retrieved from PDB with the PDB ID: 2WS2 with a resolution of 2.1 Å. The structure was prepared by removing any water molecules and adding necessary hydrogen atoms by utilizing the UCSF Chimera 1.17 to optimize the protein structure for further analyses.

### 2.2. Pharmacophore Modeling and Virtual Screening

Ligand-based pharmacophore modeling was performed by utilizing LigandScout 3.1 and its ligand-based module. Various pharmacophoric sites including hydrophobic sites, hydrogen bond acceptors and donors, aromatic rings, and negative and positive groups were considered and analyzed. All the characteristics of the drugs were incorporated, and the atom overlap scoring function and the merge feature model generation option were applied during ligand-based pharmacophore modeling. The MolPort natural compound library, containing 11,3000 compounds, was used for high-throughput virtual screening (HTVS) to shorten the search time.

### 2.3. Lead Compound Docking

The energy minimization and geometric optimization of the selected compounds were performed through UCSF Chimera 1.17.3 and ChemDraw Ultra. The molecular docking analyses were performed by employing AutoDock Vina and PyRx. The molecular docking analyses were conducted to evaluate the binding orientations and identify optimal binding conformations. UCSF Chimera 1.17, Discovery Studio, and Ligplot were employed to analyze and visualize the binding interactions.

### 2.4. ADMET Analysis

The adsorption, distribution, metabolism, excretion and toxicity (ADMET) properties of the scrutinized compounds were evaluated by using the admetSAR online server to calculate the possible mutagenic and carcinogenic risks. The drug-like properties of the selected compounds were also calculated according to Lipinsky’s Rule of Five [33].

### 2.5. Molecular Dynamics (MD) Simulations

Schrödinger LLC (New York, NY, USA) embedded software Desmond was used to perform molecular dynamics (MD) simulations for 100 nanoseconds (ns) [34,35,36]. The MD simulation study was employed to analyze the ligand-binding pattern in the physiological environment by incorporating Newton’s classical equation of motion.

The selected complexes of ligands and proteins were minimized and optimized by using Maestro’s Protein Preparation Wizard. The distorted geometries, bad contacts, and steric clashes were removed prior to MD simulation analyses. The system was built by using the System Builder tool, and an orthorhombic box of Intermolecular Interaction Potential 3 Points Transferable (TIP3P) was utilized as a solvent model with the OPLS_2005 force field [37]. The system was neutralized by adding appropriate counter ions to the models, and 0.15 M of sodium chloride was added to simulate the physiological conditions with 1 atm pressure and 300 K temperature throughout the simulation period [38,39,40]. For a detailed analysis, the generated trajectories were saved after every 110 picoseconds (ps). Moreover, the stability of the protein–ligand complex was validated through root mean square deviation (RMSD) over time.

## 3. Results

**Figure 1:**
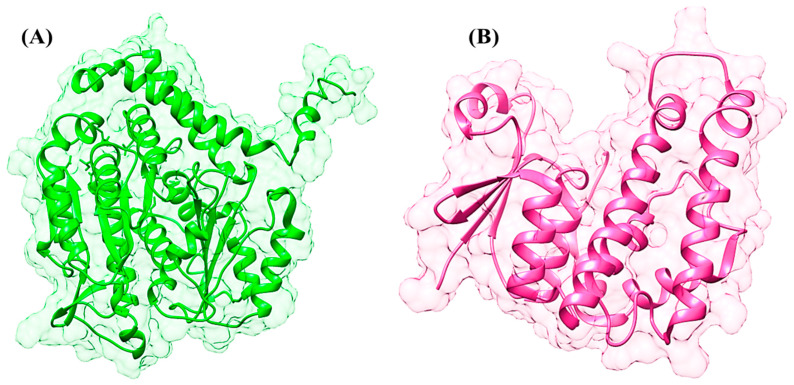
(**A**) The 3-dimensional structure of beta-tubulin isotype-1. (**B**) The 3-dimensional structure of glutathione S-transferase.

The 3D structure of GST was retrieved from PDB; however, the 3D structure of β-tubulin isoform 1 was predicted through different in silico approaches. The 3D structure of β-tubulin isoform 1 has not been identified by NMR and X-ray crystallography techniques. Threading, ab initio, and comparative modeling approaches were employed to predict the 3D structures of β-tubulin isoform 1. The retrieved amino acid sequence of β-tubulin isoform 1 was subjected to protein BLAST for suitable templates against the PDB database. The ten optimally aligned, top-ranked suitable templates, with total scores, E values, maximum identity, and query coverage, were selected to predict the 3D structures through a homology modeling approach. It was observed through the sequence alignment of the protein residues that the evolutionarily conserved region of the sequence would show similar functions. All the selected templates were used to predict the 3D structure of β-tubulin isoform 1. However, it was observed that the similarity and overall query coverage among all the selected templates was 98% from end to end, which was not considered reliable for 3D structural prediction through homology modeling. Ab initio and threading approaches were used to overcome the errors and to produce a reliable 3D structure of β-tubilin isoform 1. Numerous structures of beta-tubulin isoform 1 were predicted by employing different tools satisfying the sequence. All the predicted structures were further evaluated based on the overall quality factor, allowed region, favored region, outliers, and binding regions. The results revealed that the structures predicted through the threading approach were more reliable (Figure 1A). The overall quality factor of 90.90%, favored region of 79.1%, allowed region of 18.6%, and outliers of 0.3% were observed in the predicted structure depicting the high quality of the predicted structure. The energy minimization was performed on the predicted structure to improve the stereochemistry, for minimization at 1000 steepest descent and conjugate gradient steps. These parameters revealed that the selected minimized structure had the potential for further analyses.

The 3D structure of GST (PDB ID: 2WS2) was retrieved from the PDB for in silico drug design investigations. The retrieved structure was processed and all the missing residues were predicted, minimized, and optimized. Loop refinement was performed, an adequate number of hydrogen atoms was added, and a bond order was assigned to produce the native conformation of the selected protein (Figure 1B).

An extensive literature review revealed that anthelmintic compounds have potential activity against *Haemonchus contortus,* and 12 anthelmintic compounds were selected (Table 1) for the current analysis. The molecular docking studies of the selected anthelmintic compounds showed variations in their binding energies. A total of 150 runs were performed for each molecular docking analysis. Interactional analyses were performed, binding pockets were identified through the lowest binding energy, and repeated binding residues for each drug compound were observed. It was observed that the selected twelve anthelmintic compounds effectively bound to both the selected target proteins and showed potent binding residues (Table 1).

Molecular docking analyses were employed of the selected anthelmintic compounds. The results revealed that all the anthelmintic compounds bound to a similar binding pocket of the selected target proteins.

The ligand-based pharmacophore was generated by using the selected anthelmintic compounds. Various pharmacophoric sites (hydrogen bond donor (HBD) and acceptor (HBA), hydrophobic sites, aromatic ring, positive and negative ionizable groups) were carefully characterized. The merge-feature model generation and atoms overlap scoring function were applied to represent the characteristics of selected anthelmintic compounds. The MolPort natural compound library, containing 113,000 compounds, was screened and the top-ranked 500 compounds were selected.

Molecular docking analysis was performed, and complexes were ranked on the bases of drug properties, highest binding affinity, and lowest binding energy. The top-ranked 100 docked complexes were analyzed. Interestingly, it was observed that the screened compounds showed the lowest binding energies (Table 2) and potently bonded to a similar binding site, as observed in the docking analyses of selected anthelmintic compounds. The screened natural compounds showed the lowest binding energy against the target proteins compared to other anthelmintic compounds.

All the screened compounds and 12 selected anthelmintic compounds bound to the same binding site of the target proteins.

The screened compounds showed the least binding energy, highest binding affinity, and effective drug properties (Table 2). However, only minimal fluctuations were observed in the analyzed complexes with the lowest binding energies.

**Figure 2:**
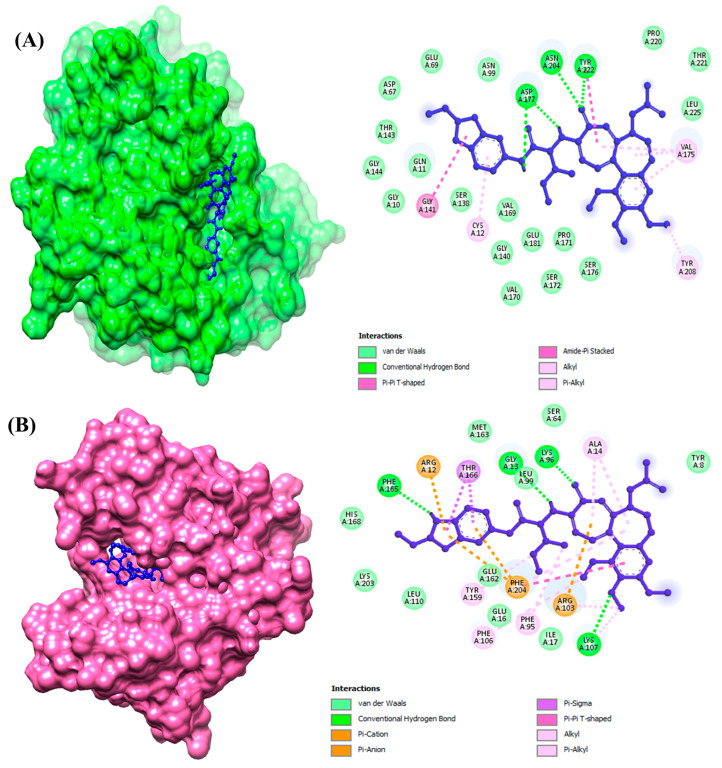
The interactional analysis of compound Molport-039-195-358 against (**A**) beta-tubulin isotype 1 and (**B**) glutathione S-transferase.

The plots of protein–ligand interactions were analyzed and visualized for a better visualization of the interactions between the residues of ligand atoms and protein amino acid residues (Figure 2). Interestingly, it was observed that the top-ranked compounds for both the selected target proteins were the same and showed the lowest binding energy along with potent drug properties.

Glu45, Arg46, Cys126, Gln131, Lys252, Asn247, and Arg251 were observed as the conserved binding residues of β-tubulin isotype 1. Leu99, Asn100, Arg103, Lys107, Glu162, and Met163 were the interacting residues of GST.

The ADMET analysis of the selected compounds revealed promising pharmacokinetic and safety characteristics (Table 3). The compounds exhibited good absorption properties and a favorable distribution with no potential to cross the blood–brain barrier, reducing the risk of central nervous system effects. The pharmacokinetic properties of the top-ranked compounds illustrated that the selected compounds are non-toxic and non-carcinogenic, which supports their potential as safe candidates for further drug development.

**Figure 3:**
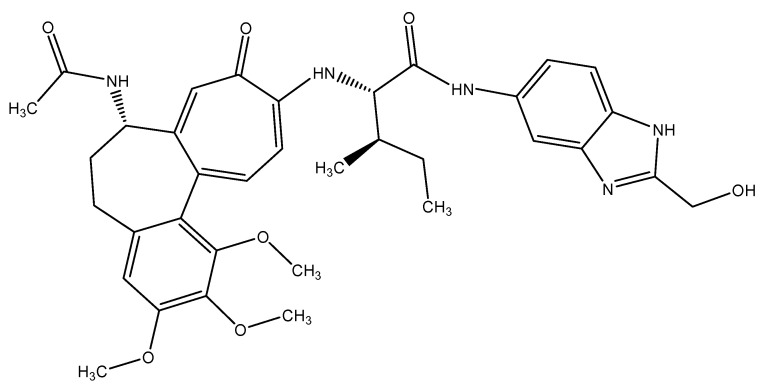
The 2-dimensional structure of Molport-039-195-358.

**Figure 4:**
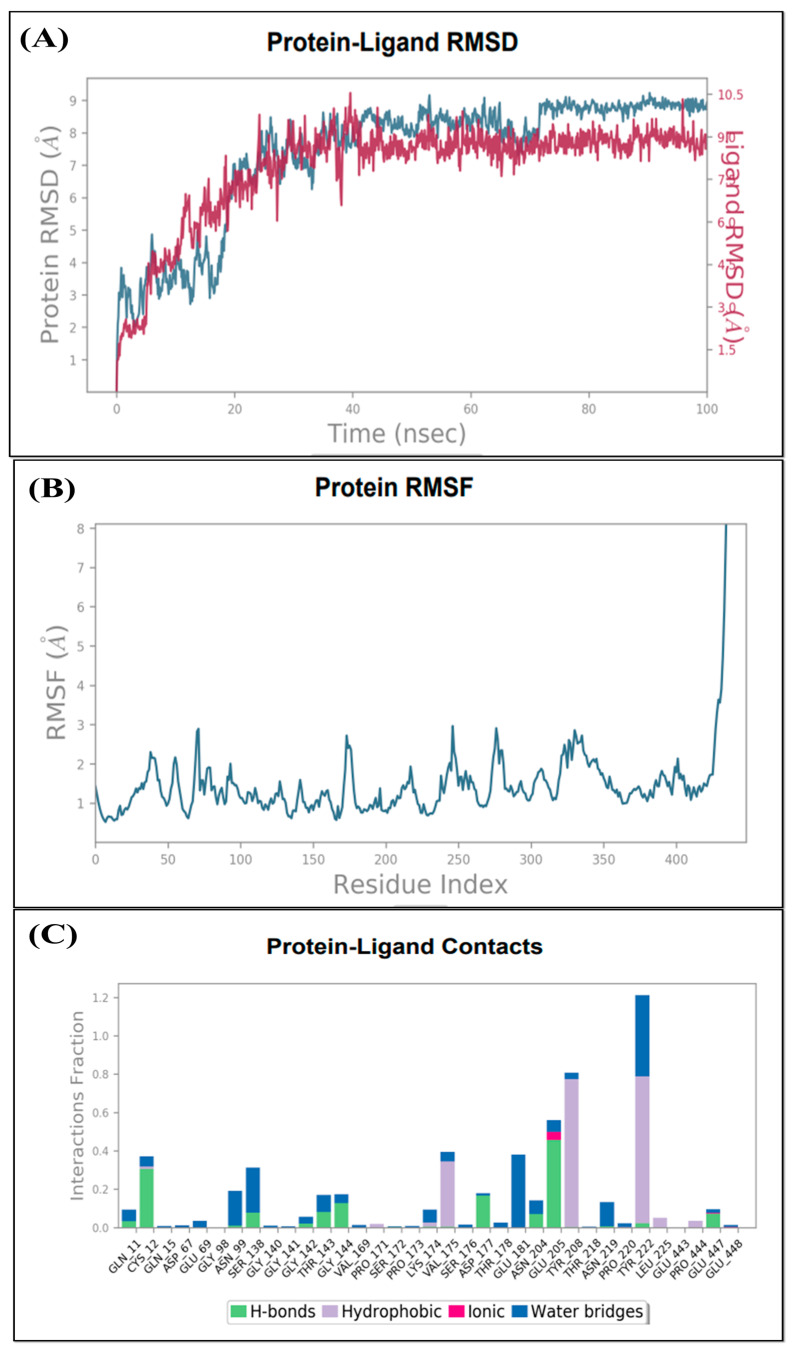
(**A**) Root mean square deviation of β-tubulin complex. (**B**) β-tubulin root mean square fluctuation. (**C**) Interacting residues of β-tubulin.

**Figure 5:**
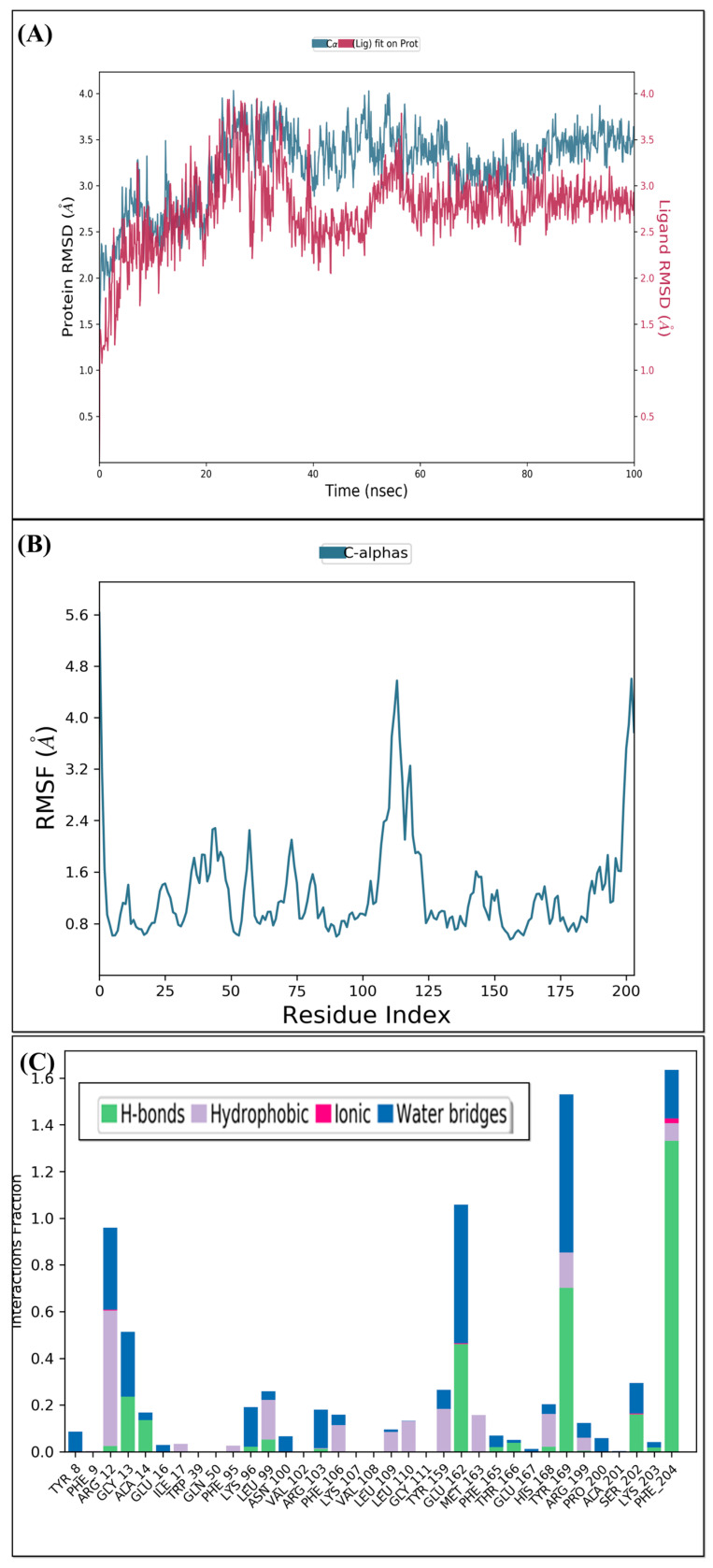
(**A**) Root mean square deviation of glutathione S-transferase. (**B**) Root mean square fluctuation of glutathione S-transferase. (**C**) Interacting residues of glutathione S-transferase.

In order to examine the stability of the top-ranked, selected compounds for both the selected target proteins. Compound Molport-039-195-358 (Figure 3) was selected for MD simulation analysis for both the selected target proteins, as Molport-039-195-358 showed promising results for both targets. The RMSD, RMSF, protein–ligand contacts, and hydrogen bond analyses were performed (Figure 4 and Figure 5). By analyzing the RMSD of proteins and ligands, it was observed that both complexes of selected target proteins showed an equilibrium with the passage of time and exhibited lower RMSD values, depicting the stability of the complexes. The stability of the backbone of both the target proteins was observed throughout the simulation period. Both the complexes had an average RMSD of 0.233 nm, which illustrates that the protein backbone remained highly stable upon ligand binding. Furthermore, the RMSF values of both the selected proteins with compound Molport-039-195-358 were analyzed carefully. It was observed that all protein residues exhibited low RMSF values. In the β-tubulin isotype 1 complex with Molport-039-195-358, residues such as TYR222, GLU225, and ASN205 were forming hydrogen bonds, which also contributed to the stability. In the GST complex, ARG-12, GLY-13, GLU-162, TYR-169, and PHE-204 were forming hydrogen bonds, thereby stabilizing the complex.

## 4. Discussion

The current study focuses on identifying the inhibitors of two proteins (glutathione S-transferase (GST) and β-tubulin isotype-1) of the parasite *Haemonchus contortus,* which cause haemonchosis in small ruminants, leading to significant economic losses from morbidity and mortality [41,42,43]. The resistance of *H. contortus* to anthelmintic drugs including benzimidazoles and ivermectin has made it crucial to look for alternative treatments [44]. Moreover, other classes of drugs exhibited different mechanisms. Macrocyclic lactones including ivermectin target the channels of glutamate-gated chloride that induce paralysis. Imidazothiazole (levamisole) behaves as an agonist at nicotinic acetylcholine receptors and also causes paralysis. In the present study, extensive in silico analysis was performed to identify the natural compounds with inhibitory potential against target proteins. The findings provide valuable insights into potential new therapeutic strategies for controlling haemonchosis in livestock.

In silico analysis, structural bioinformatics including molecular docking, and virtual screening help to identify potent compounds by predicting the ADMET properties, binding affinity, binding energy, and molecular interactions. Molecular docking studies are considered an important approach to identify the active compounds against different disease-related proteins [45]. Molecular docking analysis was performed for the target proteins against the known anthelmintic compounds to identify the binding pocket. The interactional analysis revealed that the conserved residues (Glu45, Arg46 and Cys126 in beta-tubulin, and Leu99, Arg103, and Glu162 in GST) showed the promising region for binding. The top-ranked compounds from molecular docking analysis were used for pharmacophore modeling. Ligand-based pharmacophore screening was performed to identify the natural compounds with more promising features in comparison with the synthetic anthelmintic compounds. The top-ranked 200 compounds resulting from the virtual screening were separately docked with both the targets. Compound 534,313 and compound Molport-039-195-358 showed promising binding affinities, with the lowest binding energies of −9.4 kcal/mol and −9.5 kcal/mol against β-tubulin isoform1 and GST, respectively.

Unlike earlier studies that focused on identifying inhibitors through experimental methods which can be time-consuming, we used in silico analysis, because the in silico approach offers an efficient strategy for drug discovery. The findings of current studies indicated that the reported compounds showed better results as compared to the control. GST and β-tubulin isotype 1 were reported as therapeutic targets due to their role in drug resistance and cellular functions in *H. contortus* [5,10]. Natural compounds such as glycyrrhetinic acid and thymol have been reported as potential inhibitors of beta-tubulin isotype 1 from drug-resistant *H. contortus*; however, their use is limited [11]. The inhibitors of GST were also reported through in vitro and in silico studies [9]. Interestingly, the findings of the present study are in line with the reported results. The current study identified a single compound against two different target proteins through extensive in silico analyses. Compound Molport-039-195-358 is a dual target inhibitor of GST and beta-tubulin isoform1 identified by the in silico approach. These findings are novel and provide deep insights about the development of a safer and effective therapeutic intervention to control haemonchosis in animals for profitable livestock production.

## 5. Conclusions

The present study successfully identified potential natural inhibitors targeting GST and β-tubulin isotype-1 in *Haemonchus contortus* using in silico approaches. The findings revealed that Molport-039-195-358 demonstrated high binding affinities and stability, suggesting its potential to disrupt essential parasite functions and combat drug resistance. ADMET analysis confirmed its drug-likeness and safety, providing a foundation for developing novel anthelmintic agents. This computational strategy offers a cost-effective and efficient pathway for discovering new therapeutics to control haemonchosis in livestock, addressing the critical issue of drug resistance in the livestock industry. However, further in vitro and in vivo trials are warranted to validate the efficacy of the identified compound in future studies. 

## Figures and Tables

**Table 1:** The binding energies of previously reported anthelmintic compounds with selected target proteins.

Compounds ID	With Beta-Tubulin Isotype-1 (Kcal/mol)	With GST (Kcal/mol)	Binding Residues (β-Tubulin Isotype-1)	Binding Residues (GST)
9832750	−8.7	−10.4	Arg2, Glu45, Arg46, Cys129, Gln131, Cys239, Phe242, Pro243, Gly-44, Asp249, Arg251, Lys252	Tyr8, Gly13, Ala14, Gln63, Val65, Phe95, Lys96, Leu99, Asn100, Arg103, Phe106, Lys107, Glu162, Met163, Phe204
135449328	−7.4	−8.9	Gly10, Gln11, Cys12, Gln131, Ser138, Gly144, Asp177, Thr178, Tyr222, Leu225	Arg12, Gly13, Glu16, Ile17, Phe95, Leu99, Arg103, Phe106, Lys107, Leu110 Try159, Glu162, Met163, Thr166, His168, Ser202, Phe204
4030	−7.8	−8.2	Gln11, Cys12, Asn99, Ser138, Leu139, Gly140, Gly141, Val178, Asp177, Glu181	Arg12, Gly13, Ala14, Glu16, Ile17, Ser64, Phe95, Leu99, Arg103, Phe106, Lys107, Leu110 Try159, Glu162, Met163, Phe165, Thr166, His168, Lys203, Phe204
40854	−7.5	−7.8	Cys12, Ala97, Asn99, Ser138, Leu139, Gly140, Gly141, Val169, Val175, Val178, Asp177	Arg12, Gly13, Phe95, Arg103, Phe106, Lys107, Leu110 Try159, Glu162, Met163, Phe165, Thr166, His168, Lys203, Phe204
71449	−6.7	−7.8	Gly10, Gln11, Cys12, Ala97, Asn99, Ser138, Leu139, Gly140, Gly141, Val169, Val175, Val178, Asp177, Asn204	Arg12, Gly13, Ala14, Phe95, Leu99, Arg103, Phe106, Lys107, Leu110 Try159, Glu162, Met163, Phe165, Thr166, His168, Phe204
50248	−6.6	−7.5	Gln11, Cys12, Asn99, Ser138, Leu139, Gly140, Gly141, Val178, Asp177, Glu181	Arg12, Gly13, Ala14, Glu16, Ile17, Ser64, Phe95, Leu99, Arg103, Phe106, Lys107, Leu110 Try159, Glu162, Met163, Thr166, His168
83969	−6.9	−7.2	Gly10, Gln11, Cys12, Glu69, Ala97, Gly98, Asn99, Ser138, Leu139, Gly140, Gly141, Val169, Val175, Val178, Asp177, Asn204	Arg12, Gly13, Glu16, Ile17, Phe95, Leu99, Arg103, Phe106, Lys107, Leu110 Try159, Glu162, Met163, Phe165, Thr166, His168, Phe204
33309	−7	−7.1	Cys12, Glu69, Thr72, Asn99, Ser138, Leu139, Gly140, Gly141, Val170, Pro171, Val175, Val178, Asp177, Asn204	Arg12, Gly13, Ala14, Phe95, Leu99, Arg103, Phe106, Lys107, Try159, Glu162, Met163, Phe165, Thr166, Phe204
4622	−6.3	−7.1	Gln11, Cys12, Ala97, Asn99, Ser138, Leu139, Gly140, Gly141, Val169, Val175, Val178, Asp177, Asn204	Arg12, Gly13, Glu16, Ile17, Phe95, Leu99, Arg103, Phe106, Lys107, Leu110 Try159, Glu162, Met163, Phe165, Thr166, His168, Phe204
2082	−6.3	−7	Gly10, Gln11, Cys12, Ala97, Asn99, Ser138, Leu139, Gly140, Gly141, Val169, Val175, Val178, Asp177	Arg12, Gly13, Glu16, Ile17, Phe95, Leu99, Arg103, Phe106, Lys107, Leu110, Leu158, Try159, Glu162, Met163, Thr166
26879	−5.8	−6.9	Gln11, Cys12, Ser138, Leu139, Gly140, Gly141, Val178, Asp177, Glu181	Arg12, Gly13, Glu16, Ile17, Phe95, Try159, Glu162, Met163, Thr166
5430	−6.2	−6.7	Gln11, Cys12, Asn99, Ser138, Leu139, Gly140, Gly141, Val169, Val175, Val178, Asp177, Asn204	Arg12, Gly13, Glu16, Ile17, Phe95, Try159, Glu162, Met163, Thr166, Phe204

**Table 2:** Binding energies of top ranked compounds with β-tubulin isotype-1 and glutathione S-transferase.

Compounds	With β-Tubulin Isotype-1 (Kcal/mol)	With GST (Kcal/mol)
Molport-000-534-313	−9.4	−9.4
Molport-039-195-358	−9.4	−9.5
Molport-000-534-195	−8.7	−9.0
Molport-000-534-076	−10.2	−8.4
Molport-000-097-781	−9.1	−8.7
Molport-000-534-254	−8.9	−8.7
Molport-000-532-863	−8.7	−9.7
Molport-000-092-797	−8.8	−8.8
Molport-002-594-703	−7.8	−9.7
Molport-000-532-878	−8.8	−7.9

**Table 3:** Adsorption, distribution, metabolism, excretion, and toxic property calculations for top-ranked compound Molport-039-195-358.

Model	Result	Probability
Adsorption		
Blood–Brain Barrier	BBB−	0.8433
Human Intestinal Absorption	HIA+	0.9661
Caco-2 Permeability	Caco-2	0.7124
P-glycoprotein Substrate	Substrate	0.7854
P-glycoprotein Inhibitor	Non-inhibitor	0.9112
Distribution		
Subcellular Localization	Nucleus	0.4909
Metabolism		
CYP450 2C9 Substrate	Non-substrate	0.7745
CYP450 2D6 Substrate	Non-substrate	0.8307
CYP450 1A2 Inhibitor	Non-inhibitor	0.6984
CYP450 2C9 Inhibitor	Non-inhibitor	0.6149
CYP450 2D6 Inhibitor	Non-inhibitor	0.8617
CYP450 2C19 Inhibitor	Non-inhibitor	0.7318
CYP450 3A4 Inhibitor	Non-inhibitor	0.7707
Toxicity		
AMES Toxicity	Non-AMES toxic	0.6349
Carcinogens	Non-carcinogens	0.9172

## Data Availability

All data generated during this study are available within the manuscript.
